# Gliadin Peptide P31-43 Localises to Endocytic Vesicles and Interferes with Their Maturation

**DOI:** 10.1371/journal.pone.0012246

**Published:** 2010-08-18

**Authors:** Maria Vittoria Barone, Merlin Nanayakkara, Giovanni Paolella, Mariantonia Maglio, Virginia Vitale, Raffaele Troiano, Maria Teresa Silvia Ribecco, Giuliana Lania, Delia Zanzi, Sara Santagata, Renata Auricchio, Riccardo Troncone, Salvatore Auricchio

**Affiliations:** 1 Pediatric Department and European Laboratory for the Investigation of Food-Induced Disease (ELFID), University of Naples Federico II, Naples, Italy; 2 CEINGE–Biotecnologie Avanzate, Naples, Italy; 3 Biochemistry Department, University of Naples, Federico II, Naples, Italy; Iowa State University, United States of America

## Abstract

**Background:**

Celiac Disease (CD) is both a frequent disease (1∶100) and an interesting model of a disease induced by food. It consists in an immunogenic reaction to wheat gluten and glutenins that has been found to arise in a specific genetic background; however, this reaction is still only partially understood. Activation of innate immunity by gliadin peptides is an important component of the early events of the disease. In particular the so-called “toxic” A-gliadin peptide P31-43 induces several pleiotropic effects including Epidermal Growth Factor Receptor (EGFR)-dependent actin remodelling and proliferation in cultured cell lines and in enterocytes from CD patients. These effects are mediated by delayed EGFR degradation and prolonged EGFR activation in endocytic vesicles. In the present study we investigated the effects of gliadin peptides on the trafficking and maturation of endocytic vesicles.

**Methods/Principal Findings:**

Both P31-43 and the control P57-68 peptide labelled with fluorochromes were found to enter CaCo-2 cells and interact with the endocytic compartment in pulse and chase, time-lapse, experiments. P31-43 was localised to vesicles carrying early endocytic markers at time points when P57-68-carrying vesicles mature into late endosomes. In time-lapse experiments the trafficking of P31-43-labelled vesicles was delayed, regardless of the cargo they were carrying. Furthermore in celiac enterocytes, from cultured duodenal biopsies, P31-43 trafficking is delayed in early endocytic vesicles. A sequence similarity search revealed that P31-43 is strikingly similar to Hrs, a key molecule regulating endocytic maturation. A-gliadin peptide P31-43 interfered with Hrs correct localisation to early endosomes as revealed by western blot and immunofluorescence microscopy.

**Conclusions:**

P31-43 and P57-68 enter cells by endocytosis. Only P31-43 localises at the endocytic membranes and delays vesicle trafficking by interfering with Hrs-mediated maturation to late endosomes in cells and intestinal biopsies. Consequently, in P31-43-treated cells, Receptor Tyrosin Kinase (RTK) activation is extended. This finding may explain the role played by gliadin peptides in inducing proliferation and other effects in enterocytes from CD biopsies.

## Introduction

Celiac disease (CD) is characterised by a derangement of both the adaptive and the innate immune response to gliadin. Some gliadin peptides that are deamidated by tissue transglutaminase (e.g., A-gliadin P57-68) bind to HLA DQ2 and/or DQ8 molecules [1] and induce an adaptive Th1 proinflammatory response. In the case of the innate immune response, [2] A-gliadin P31-43, which is not recognised by T cells, [3], [4] induces IL15 production, which in turn is thought to cause expansion of intra epithelial lymphocytes (IEL) in CD and epithelial apoptosis. [5–6–7] Furthermore, IL15 has been implicated in the increased expression of NKG2D on lymphocytes. The interaction between the major histocompatibility complex (MHC) class I chain-related gene A (MICA), and NKG2D is at least in part responsible for IEL-induced enterocyte apoptosis and villous atrophy. [8]–[9]

Many biological activities have been associated with gliadin peptides in several cell types [10–11–12–13–14] including reorganisation of actin and increased permeability in the intestinal epithelium. [15]–[16] Other effects are specific to celiac tissues. In untreated celiac patients, P31-43 prevented the restitution of enterocyte height, which normally occurs after 24–48 h of culturing mucosal explants with medium alone. [17] P31-43 damaging activity has been demonstrated in organ culture of treated celiac biopsies, [18] and in *in vivo* feeding studies. [19] Similar results have been obtained *in vivo* on small intestinal and oral mucosa with the A-gliadin peptide 31–49. [20]–[21]

It has yet to be established to what extent these properties relate to the ability of these A-gliadin peptides to activate innate immunity mechanisms. Virtually nothing is known about the mechanisms underlying the biological properties of P31-43 or about the metabolic pathways involved in the activation of innate immunity in CD. Similarly, it is not known why celiac patients are particularly sensitive to these biological activities.

We recently investigated the molecular basis of the non-T cell-mediated properties of the gliadin peptides most likely to play an important role in the very early phases of CD, and we found that P31-43 causes actin alterations and cell proliferation, both of which depend on activation of the epidermal growth factor receptor (EGFR), in several cell types, and in the organ culture of celiac mucosa. [22]–[23] In this system P31-43 interferes with EGFR decay and prolongs EGFR activation. We also showed that P31-43 increases IL15 on the cell surface, by interfering with its trafficking (MV Barone, unpublished data). These data suggest that enhancement of EGFR and IL15 signalling may be important biological contributors to the pathogenesis of CD.

Here we demonstrate that both P31-43 and P57-68 enter CaCo 2 cells and interact with endocytic compartment, but only P31-43 interferes with the endocytic pathway by delaying maturation of early endosomes to late endosomes. We also show that the P31-43 sequence is similar to hepatocyte growth factor-regulated tyrosine kinase substrate (Hrs), which is a key protein of endocytic maturation. [24] P31-43 is localised at the vesicles membranes and interferes with the correct localisation of Hrs to endocytic vesicles thus delaying the maturation of early endosomes to late endosomes. Consequently the activation of EGFR and other receptors is expanded with multiple effects on various metabolic pathways and cellular functions.

## Materials and Methods

### Cell culture, materials and transfections

xCaCo-2 cells were grown in Dulbecco's Modified Eagle's Medium (DMEM) (GIBCO, San Giuliano Milanese, Italy), 10% foetal calf serum (FCS) (GIBCO, San Giuliano Milanese, Italy), 100 units/ml penicillin-streptomycin (GIBCO, San Giuliano Milanese, Italy), and 1 mM glutamine. Lipopolysaccharide-(LPS) free synthetic peptides [22] (Inbios, Naples, Italy, >95% pure, MALDI-TOFF analysis, as expected) were obtained by Ultrasart-D20 filtration (Sartorius AG, Gottingen, Germany). Levels of LPS were undetectable (<0.20 EU/mg as assessed with a commercial kit: QCL-1000 kit, Cambrex Corporation, NJ). The P31-43 sequence is LGQQQPFPPQQPY; and the P57-68 sequence is QLQPFPQPQLPY. The labelled peptides were produced as the unlabelled peptides. Solutions were used in the following concentrations: P31-43-lissamine (liss), P31-43 CY3 and P57-68-liss at 20 micrograms/ml: unlabeled peptides were used as previously reported, [22] at 70 micrograms/ml; EGF at 100 nanograms/ml; EGF-Alexa-488 at 20 nanograms/ml (Molecular Probes, San Giuliano Milanese, Italy); Dextran-Alexa488 (MW 10000) (Molecular Probes, San Giuliano Milanese, Italy) at 0.5 milligrams/ml; goat polyclonal antibody against EEA1 (C-15) at 2 micrograms/ml (Santa Cruz, DBA, Milan, Italy); mouse monoclonal antibody against LAMP2 (H4B4) at 2 micrograms/ml (Santa Cruz, DBA, Milan, Italy); secondary antibodies anti goat-Alexa-488 conjugated (Molecular Probes) for EEA1 staining at a ratio of 1∶100; and anti mouse-Alexa 488 conjugated (Molecular Probes) for LAMP2 staining at a ratio of 1∶100. Rab5-EGFP and Rab7 EGFP were kindly provided by Prof. M. Zerial (Max Planck Institute of Molecular Cell Biology and Genetics, Dresden, Germany) and Hrs-EGFP was kindly provided by Prof. P.P. Di Fiore (Fondazione Istituto FIRC di Oncologia Molecolare, Milan, Italy).

### Transfections and BrdU incorporation

We used the lipofectamine kit (Invitrogen, San Giuliano Milanese, Italy) according to the manufacturer's instructions to transfect all plasmids (Rab5-EGFP, Rab7-EGFP, Hrs-EGFP and Hrs-Ha). Briefly, CaCo-2 cells seeded on coverslips for 48 h were transfected with the plasmids for 16 h. The next day, transfected cells were pulsed and chased as described below or stained for Bromodeoxyuridine (BrdU) with a monoclonal antibody (Sigma-Aldrich, Milan, Italy) and total nuclei were identified by Hoechst staining. BrdU incorporation was performed as described elsewhere [22], briefly, CaCo-2 cells seeded on coverslips were transfected with Hrs-EGFP and serum starved in DMEM 0.1% foetal calf serum, antibiotics and glutamine for 30–48 h followed by 24 h treatment with gliadin peptides and/or growth factors. BrdU (100 mM, Boehringer) was added for one hour before fixing the samples. The cells fixed with paraformaldehyde and permeabilised with triton, were stained for BrdU as described [22] and observed at the miscroscope (Axiophot microscope, Carl Zeiss MicroImaging, Inc.). Greater than 100 HRS-EGFP positive cells in several fields were evaluated for BrdU incorporation in each sample. The number of HRS-EGFP/BrdU positive cells was expressed as a proportion of the total Hoechst positive nuclei.

### Pulse and chase experiments

In pulse and chase experiments, transfected and untransfected cells were pulsed for 30 minutes at 37°C with a mixture of labelled and unlabeled peptides to avoid an excess of fluorochromes in the medium. Overall 20 micrograms/ml of P31-43-liss, P31-43-CY3 and P57-68-liss and 50 micrograms/ml of unlabeled peptides were used to reach the working concentration of 70 micrograms/ml. [22] The mixtures of labelled and unlabeled peptides were called P31-43-liss, P31-43-CY3 and P57-68-liss. After a 30-minutes treatment with the peptides mixtures (pulse), cells were washed five times with complete medium to eliminate fluorochrome excess. Unlabeled peptides (70 micrograms/ml) were added to the cells and incubation was continued for 3 h at 37°C (chase). Transfected coverslips were briefly fixed (5 minutes) with paraformaldehyde 3% (Sigma-Aldrich) at room temperature, then mounted. Untransfected coverslips treated with labelled peptides were washed and stained for EEA1 and LAMP2.

### EEA1 and Lamp staining

CaCo-2 cells seeded on glass coverslips were stained for 1 h at room temperature with anti-EEA1 or -LAMP2 antibody after fixation with 3% paraformaldehyde for 5 min at room temperature and mild permeabilisation with 0.2% Triton (Biorad, Milan, Italy) for 3 min at room temperature. Secondary antibodies Alexa-488 conjugated (Invitrogen) anti-goat for EEA1 and anti-mouse for LAMP2 were added to the coverslips for 1 h at room temperature. Control panels for EEA1 staining with P31-43-liss and non specific anti-goat antibody together with secondary antibodies Alexa-488 conjugated anti-goat, did not show any cross-excitation of fluorochromes (Figure S1). Similar results were obtained for Lamp2 and P56-68-liss (not shown). The coverslips were then mounted on glass slides and observed by confocal microscope (LSM 510 Zeiss). In total 40 to 50 cells were observed in each sample. Images were generated with the same confocal microscope. Co-localisation analysis was performed with AIS Zeiss software. Magnification of the micrographs was the same for all the figures shown (63× objective) unless stated differently in the legends.

### Time-lapse experiments

Cells were seeded on glass-bottom dishes (3 cm in diameter obtained from Falcon Becton Dickinson Labware; La Pont de Claix, France) to allow live observation, and they were kept in a specially designed incubator (OXO-lab, Naples, Italy) that controls temperature and CO2. After treatment with gliadin peptides, cells were observed by confocal microscopy for 10 min, during which sets of frames were acquired at 30-seconds intervals. The image stack was analysed with the help of a program that allows to record the tracks followed by individual particles [25]. Using this methodology the subsequent positions of each vesicle was identified by the observer, who, with the help of a pointer on the computer screen, stored the coordinates in a text file. The program calculated the distance and direction for each time step; the values shown are the average speed of each vesicle during the observation period. The list of coordinates was also used to draw the paths covered by the vesicles, which were superimposed onto the confocal images.

### Co-localisation analysis

Samples were examined with a Zeiss LSM 510 laser scanning confocal microscope. We used Argon/2 (458, 477, 488, 514 nanometers) and HeNe1 (543 nanometers) excitation lasers, which were switched on separately to reduce cross-talk of the two fluorochromes. The green and the red emissions were separated by a dichroic splitter (FT 560) and filtered (515-to 540-nm band-pass filter for green and >610-nm long pass filter for red emission). A threshold was applied to the images to exclude about 99% of the signal found in control images. The weighted co-localisation coefficient represents the sum of intensity of co-localising pixels in channels 1 and 2 as compared to the overall sum of pixel intensities above threshold. This value could be 0 (no co-localisation) or 1 (all pixels co-localise). Bright pixels contribute more than faint pixels. The co-localisation coefficient represents the weighted co-localisation coefficients of Ch1 (red) with respect to Ch2 (green) for each experiment. [26]–[27] The image collection and exposure times were identical for the two peptides.

### Data bank analysis

Swissprot, Trembl and InterPro data banks were searched for sequences matching peptide P31-43 and P57-68 by using Blast and FastA. Sequence alignment was performed by using ClustalW and visualized by PrettyPlot from the EMBOSS suite.

### Immunoblotting and subcellular fractionation

Near-confluent Caco2 cells in a 90-mm dish were incubated with EGF and P31-43 at various times, after homogenization (10 mM Tris-HCl [pH 7.4], 1 mM EDTA, and the mixture of phosphatase and protease inhibitors) the nuclear fraction was eliminated by centrifugation. The soluble cytosolic and the membrane fraction were obtained by ultracentrifugation. Electrophoresis and immunoblotting were performed as described elsewhere. [22] Briefly the proteins of the soluble cytosolic and the membrane fractions were separated by SDS-PAGE and incubated with anti-Hrs mouse monoclonal antibody (Alexis, Vinci-Biochem, Florence, Italy) or anti-EGFR rabbit polyclonal antibody (Cell Signaling Celbio, Milan Italy) or anti-tubulin mouse monoclonal antibody (SIGMA-Aldrich, Milan, Italy). Densitometric analysis was performed as before. [22]

### Organ culture studies

All biopsies were treated as previously described. [22] To examine the entry of P31-43-CY3 into cells, we cultured three intestinal biopsies from untreated celiac patients in the active phase of the disease and three from control subjects affected by gastro-oesophageal reflux for 3 h with P31-43-CY3 (20 micrograms/ml) and with unlabelled P31-43 (50 micrograms/ml). The samples were then washed, and chased for 24 h. Three samples from CD patients and three from controls were harvested after a 3-h pulse; three other samples from patients and controls were harvested after 24 h of chase. All samples were prepared for cryo-sectioning. Air-dried, 5 microns sections were stained for EEA1 and analysed with a confocal microscope. Anti EEA1 antibody was applied to the sections for 1 hour at room temperature and secondary antibody anti goat Alexa-488 conjugated was applied to the sections for 1 h at room temperature in a dark chamber. The protocol of the study was approved by the Ethical Committee of the University “Federico II”, Naples, Italy (Etical approval code: C.E. n. 230/05).

## Results

### Different vesicle subpopulations carry P31-43 or P57-68 peptides

Peptides P31-43 and P57-68 enter the cells and interact with vesicular compartment. We used markers of the endocytic pathway to identify the vesicular compartment that interacts with P31-43-liss and P57-68-liss.

**Figure 1:**
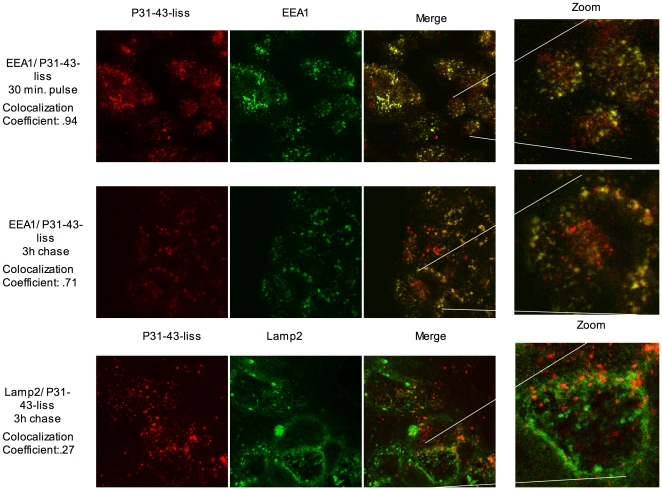
Vesicles interacting with P31-43-liss are early endocytic vesicles after a 3 h chase. In CaCo-2 cells, both after 30 minutes of pulse and 3 hours of chase with P31-43-liss (red), the peptide interacted with vesicles that are positive for EEA1 (green staining, top and middle panel). P31-43-liss did not co- localise with LAMP2-positive vesicles (green staining, bottom panel) after 3 hours of chase. A merge of the red and green panels of the EEA1 or LAMP2 and P31-43-liss or P57-68-liss staining is shown, the yellow/orange colour indicates co- localisation. The zoom panels represent a digital 4× enlargement of the region highlighted by white lines in the merge panels. The co- localisation coefficient was calculated as reported in the “Methods” section. The results are representative of four independent experiments.

**Figure 2:**
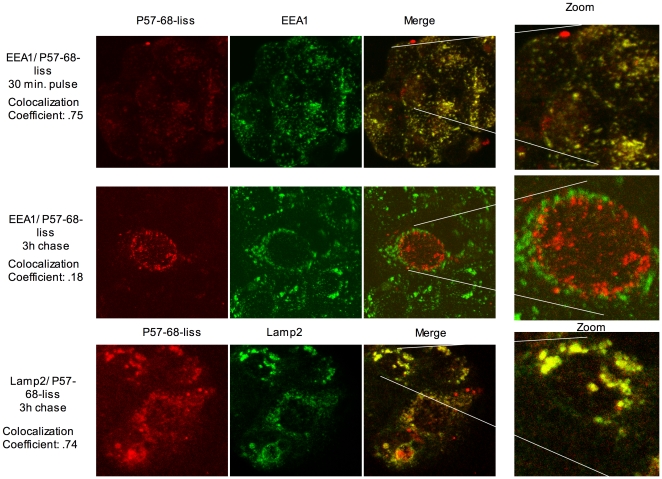
Vesicles interacting with P57-68-liss are late endocytic vesicles after a 3 h chase. At 30 minutes pulse, P57-68-liss co- localised with EEA1-positive vesicles (top panel); however after 3 hours of chase P57-68-liss no longer co- localised with EEA1-positive vesicles (middle panel). After 3 hours of chase, P57-68-liss co- localised with LAMP2-positive vesicles (bottom panel). Merge of the red and green panels of the EEA1 or LAMP2 and P31-43-liss or P57-68-liss staining is shown, yellow/orange colour indicates co- localisation. The zoom panels represent a digital 4× enlargement of the region highlighted by white lines in the merge panels. The co- localisation coefficient was calculated as reported under “Methods”. The results are representative of four independent experiments.

We used EEA1 (Figure 1) and Rab5-EGFP (not shown) as markers of early endocytosis. CaCo-2 cells treated with labelled peptides for 30 minutes (pulse) and 3 hours (chase) were stained with anti-EEA1 antibodies to identify early endocytic vesicles. Both peptides co-localised with EEA1-positive vesicles at 30 minutes (Figure 1 and 2), but only P31-43-liss-carrying vesicles were EEA1-positive after 3 hours of chase. Similar results were obtained with CaCo-2 cells overexpressing Rab 5-EGFP protein (not shown). Normal endocytic maturation requires the progression from early vesicles to late vesicles. We used the late endocytic markers LAMP2 (Figure 1) and Rab7-EGFP (not shown) to investigate the progression of endocytosis. CaCo-2 cells treated with labelled peptides for a 30-minutes pulse and a 3-hours chase were stained with anti-LAMP2 antibodies to identify late endocytic vesicles. Neither P31-43-liss nor P57-68-liss peptides co-localised with LAMP2-positive vesicles at 30 minutes (not shown). Only P57-68-liss-carrying vesicles were LAMP2-positive after 3 hours of chase (Figure 1 and 2). Taken together, these observations indicate that P31-43-containing vesicles, but not P57-68-containing vesicles are impaired in their maturation from early to late endosomes.

### P31-43-liss-carrying vesicles move more slowly than P57-68-liss-carrying vesicles

**Figure 3:**
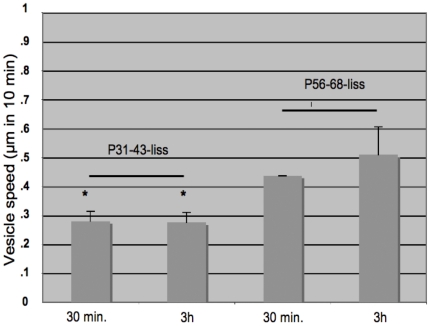
P31-43-liss-carrying vesicles are slower than P57-68-liss-carrying vesicles. Statistical analysis of three experiments performed resulted as follows: live CaCo2 cells pulsed for 30 minutes and chased for 3 hours with lissamine-labelled peptides were used for time-lapse experiments in which we acquired images every 30 seconds for 10 minutes at the indicated times. The image stacks were assembled to produce a video of vesicles dynamics. In each experiment, the position of at least 25 vesicles per cell was recorded and reconstructed to mark the trajectories of each vesicle during the observation time. The speed of vesicles was calculated by averaging the trajectories produced in 10 minutes at the indicated times. Bars represent mean and standard deviation. Asterisks indicate P<0.05 (Student's t-test). P31-43 carrying vesicles both at 30 minutes of pulse and 3 hours of chase are statistically significantly slower than P57-68 carrying vesicles.

To test the hypothesis that P31-43 could interfere with vesicle movements, we examined living cells labelled with the two peptides. The paths followed by individual vesicles were recorded after treatment with P31-43-liss and P57-68-liss peptides (Movie S1 and Movie S2). The movement of peptide-carrying vesicles was analysed for 10 minutes immediately after a 30-minutes pulse and after a 3-hours chase (Figure 3); data are reported as the average of three independent experiments. P57-68 vesicles had longer trajectories in pulse (0.44±0.009 microns/10 minutes) and chase (0.51±0.095 microns/10 minutes) experiments. P31-43-liss-interacting vesicles moved much less under both conditions (0.28±0.035 microns/10 minutes in pulse and 0.29±0.021 microns/10 minutes in chase experiments). Directionality of the endosome movements seems not to be affected by P31-43.

### P31-43-induced delay of endocytic vesicle dynamics is unrelated to their cargo

**Figure 4:**
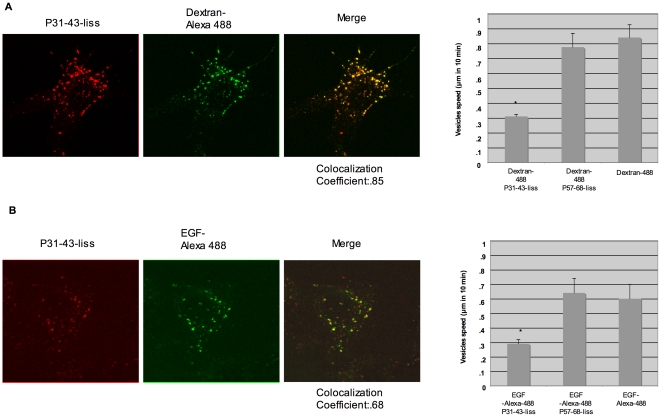
P31-43 delays endocytosis regardless of vesicles cargo. A) Dextran-Alexa-488-carrying vesicles moved faster than dextran-Alexa-488- and P31-43-liss-carrying vesicles. Live CaCo2 cells were pulsed for 30 minutes with dextran-Alexa-488 with and without P31-43-liss or P57-68-liss then chased for 3 hours in time lapse experiments. Only the 30 minutes pulse experiments are shown. The histogram shows the statistical analysis of three experiments. We calculated the speed of vesicles by averaging the trajectories produced in 10 minutes as in Figure 2. Bars represent mean and standard deviation. P31-43-liss and Dextran-Alexa-488 co-localised in the vesicular compartment. B) Live CaCo2 were pulsed for 30 minutes and chased for 3 hours (not shown) with EGF-Alexa-488 conjugated with and without P31-43-liss or P57-68-liss. Only the 30 minutes pulse experiments are shown. The histogram on the right side shows the statistical analysis of three experiments. We calculated the speed of vesicles by averaging the trajectories produced in 10 minutes as in Figure 2. Bars represent mean and standard deviation. P31-43-liss co- localises with EGF-Alexa 488 in the vesicular compartment. Asterisk indicates P<0.05 (student's t-test). The results show that P31-43 carrying vesicles are slower regardless of the cargo they are carrying.

To determine whether P31-43 is selectively directed to a specific population of slower vesicles or is able to delay endocytosis of vesicles where it is directed, we loaded CaCo-2 cells with Alexa-488 labelled dextran, a compound that is readily endocytosed in the cells, and EGF-Alexa, which normally enters the cells bound to the EGFR. CaCo-2 cells were pulsed for 30 minutes and chased for 3 hours with dextran-Alexa-488 alone or combined with P31-43-liss or P57-68 liss and then observed for 10 minutes in time-lapse microscopy. In experiments with dextran-Alexa-488 and P31-43-liss, most vesicles carry both fluorochromes, and are slower than vesicles carrying dextran alone or combined with P57-68 (Figure 4A), suggesting that the peptide, rather than the contents of the endocytic vesicle, is responsible for slower vesicle movement and delayed endocytosis.

We similarly investigated whether P31-43, which is known to interfere with EGF-carrying vesicles, [22] also affects the dynamics of EGF-Alexa-488-containing vesicles. Time-lapse analysis showed that P31-43-liss, unlike P57-68, delays EGF-Alexa-488-carrying vesicles (Figure. 4B). The fact that the peptide can delay endocytic vesicles carrying dextran or EGF strongly supports the hypothesis that the peptide is able to delay early endocytotic vesicles, regardless of the contents of the vesicles. The effects exerted by P31-43 on the endocytic pathway, in the case of EGFR, as we have shown previously, [22] result in an extension of the activation period of this receptor, which accounts for the stimulation of EGFR dependent pathways. [22] This mechanism is likely to be responsible also for activation of other receptors sharing the same endocytic pathway.

### P31-43 shares sequence similarity with Hrs, a key molecule in the maturation of endocytic vesicles

**Figure 5:**
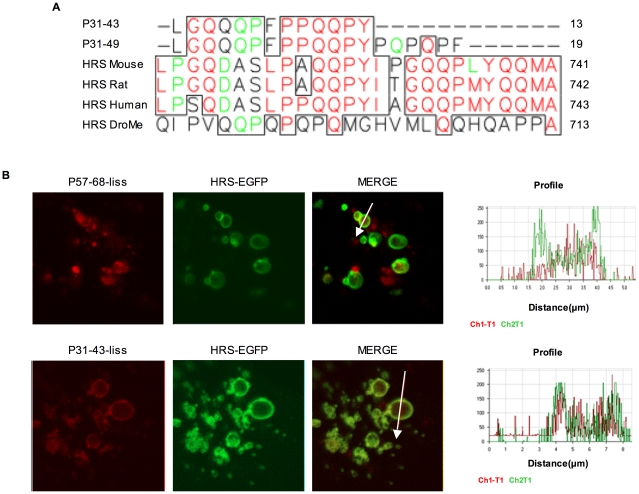
P31-43 is similar to Hrs and is localised at the membrane vesicles. A) Multiple alignments of gliadin peptides P31-43 and P31-49 are shown, with Hrs from mouse, rat, human and *Drosophila*. Numbers on the right side of the figure represent the terminal amino acid position of each sequence shown. Of the 13 residues in P31-43, 7 are identical (red) and 2 similar (green) to the corresponding residues of human Hrs. B) Enlarged vesicles visible by multiple digital enlargement (8×) of the cytosol of a single cell. Hrs-EGFP was transfected for 48 hours in CaCo2 cells. P31-43 or P57-68 was added for 15 minutes. On the right side is the profile of the red and green channels along the white arrows that run across a single vesicle in the figure. The figure is representative of four similar independent experiments. Enlargement of the endocytic vesicles allow the observation of the vesicle membrane. In a short time (15 minutes) only P31-43 but not P57-68 co- localises with Hrs-EGFP at the vesicle membrane.

We carried out a FASTA search on the SWISSPROT database using gliadin peptide 31–43 as query sequence to look for endogenous proteins with a similar primary structure. The search returned, for both P31-43 and 31–49, a match with amino acids 719–731 of human Hrs, a protein with no known relationship to gliadin. The degree of similarity is high, with a better score and e-value than many matches with gliadin family proteins. Figure 5A shows the alignment of P31-43 with human Hrs and with Hrs from mouse, rat and *Drosophila melanogaster*. Of the 13 residues in the peptide, 7 are identical and 2 similar to the corresponding residues of human Hrs, the only major difference being the peptide N-terminal leucine instead of the consensus proline. The similarity area maps within a proline/glutamine-rich domain of Hrs and is conserved better than the surrounding area among Hrs orthologs. This finding is interesting because Hrs is localised both in the cytosol and in endocytic vesicles and is involved in the transport of EGFR, PDGFR and other receptor-containing early endosomes to lysosomes. [24]–[28] We carried out the same data bank search using the gliadin peptide P57-68, and failed to find any relevant similarity with Hrs or other human proteins.

### P31-43 is localised at the level of the membranes in endocytic vesicles

We next investigated the sub-cellular localisation of gliadin peptides P31-43 and P57-68. P31-43 has been described by electron microscopy to be localized at the level of endocytic membranes. [29] When cells are transfected with Hrs, enlarged early endosomes are formed due to increased fusion; [30] furthermore detection of membrane vesicles by confocal immunofluorescence microscopy is facilitated. We then overexpressed Hrs-EGFP in CaCo2 cells for 48 h to obtain larger endosomes. P31-43 or P57-68 were added for 15 minutes and then the experiment was stopped. In Figure 5B one of these experiments is shown. As expected large endosomes can be seen with Hrs-EGFP present on the vesicles membranes. Surprisingly P31-43-liss, but not P57-68 co-localises with Hrs on the vesicles membranes, indicating that the two peptides may have different ways of entering the same endocytic vesicles.

### P31-43, but not P57-68, competes with Hrs localisation in endocytic vesicles

**Figure 6:**
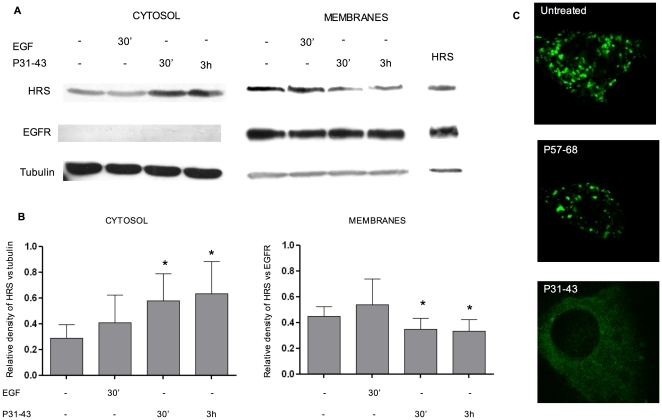
P31-43 competes with Hrs localisation to the endocytic vesicles. A) Western blot analysis of endogenous Hrs after separation by ultracentrifugation of cytosolic and membrane proteins. EGFR was used as a control for membrane fraction and tubulin as a control for cytosol. The last line on the right is a lysate of CaCo-2 cells transfected with Hrs as a size control for the protein. The results are representative of three independent experiments. B) Densitometric analysis. The Hrs concentration was normalised to a control protein, namely tubulin in the cytosolic fraction and EGFR in the membrane fraction. Mean and SD of three independent experiments is shown. Asterisk indicates P<0.05 (student's t-test). After P31-43 treatment, Hrs increases in the cytosolic fraction and decreases in the membrane fraction in comparison to the not treated sample in a statistically significant way. C) Confocal analysis of CaCo-2 cells transfected for 24 h with Hrs-EGFP, not treated and treated with P57-68 and P31-43 for the last 3 h of transfection. The results are representative of four independent experiments.

Because there is a close sequence similarity between P31-43 and Hrs within a region that is important for the localisation of Hrs to the vesicle membrane, we evaluated whether the peptide could interfere with Hrs localisation to the vesicles. To this aim, we treated CaCo-2 cells with P31-43 or EGF, and then separated cytosolic and membrane bound proteins by ultracentrifugation to quantify the amount of endogenous Hrs present in each compartment. [30] P31-43 resulted in an increase in the amount of Hrs in the cytosolic fraction, which reached a maximum 3 h after treatment (Figure. 6A,B), whereas the amount of Hrs was decreased in the membrane fraction. This effect was unrelated to the enhanced EGF pathway because EGF treatment alone did not affect Hrs concentration in either compartment, in agreement with previous results. [30] The Hrs concentration was normalised to a control protein stained on the same blot, namely, tubulin in the cytosolic fraction and EGFR in the membrane fraction. [30] We used tubulin and EGFR because they selectively localise to the cytosolic and membrane fractions and their concentration is not affected by P31-43 treatment.

The results of confocal analysis are in agreement with the results of the western blot experiment. In fact, 24 h after transfection of the Hrs-EGFP fusion protein into CaCo-2 cells, fluorescence was mostly associated with vesicles, whereas 3 h treatment with P31-43 resulted in diffused cytoplasmic staining (Figure 6C). Hrs remained associated to vesicles in the control peptide P57-68 (Figure 6C).

### Hrs overexpression prevents entry into the cell cycle induced by P31-43

**Figure 7:**
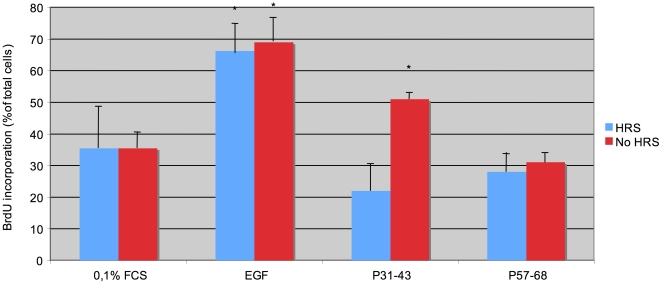
Hrs competes with the effects of P31-43 on the G0 >S transition. Bromodeoxyuridine (BrdU) incorporation of CaCo-2 cells transfected or not with Hrs-EGFP cDNA and treated as indicated 24 h after transfection. The bars represent the fraction of BrdU incorporating cells as a percent of total cells and are the mean ± SD of three independent experiments. Asterisks indicate P<0.05 (Student's t-test) with respect to the 0,1% FCS. These results indicate that Hrs compete with the effects exerted by P31-43 on cell proliferation.

Evidence suggests that gliadin peptides affect the cell cycle by delaying receptor inactivation. [22] Should this effect be due to competition of the gliadin peptide P31-43 with Hrs, large over expression of HRS cDNA would result in reversal of the effect. We therefore evaluated whether Hrs can compete with the effects induced by P31-43 on the cell cycle. As shown in Figure 7, CaCo-2 cells over expressing Hrs were stimulated to proliferate by adding EGF, P57-68 or P31-43. Under these conditions, EGF stimulation greatly increased BrdU incorporation in Hrs-transfected and untransfected CaCo-2 cells (Hrs = 66.25%±9.53%; No Hrs = 68.99%±7). As expected from a previous study, [22] P31-43 induced proliferation of non-Hrs-expressing CaCo-2 cells, with a BrdU incorporation of 51%±2% that decreased to 22.8%±8.4% in Hrs-expressing CaCo-2 cells. P57-68 did not induce proliferation. These data indicate that Hrs can compete with the effects exerted by P31-43 on cell proliferation.

### In cultured small intestine samples from biopsies, P31-43 enters the enterocytes and interacts with early endocytic vesicles

**Figure 8:**
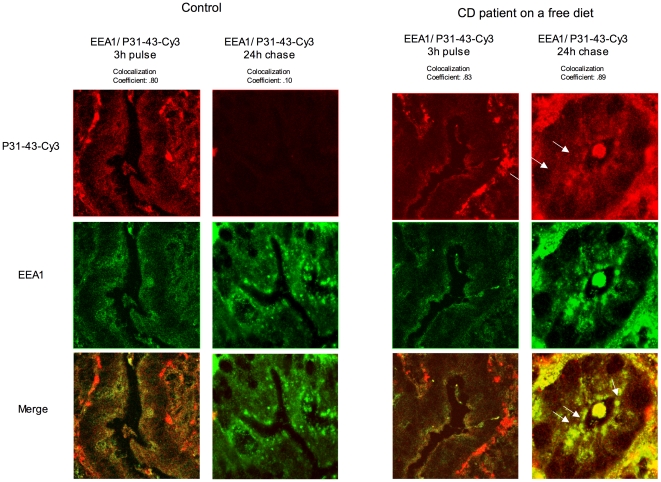
After a 24-h chase, gliadin peptide P31-43-CY3 is still present in epithelial cells of crypts of celiac disease patients, but not of controls. Intestinal biopsies from control subjects (A) and celiac disease patients on a gluten-containing diet (B) were cultivated with CY3-labelled P31-43 for 3 h and then chased for 24 h as indicated. Thin sections of the cultivated biopsies were then stained with anti-EEA1 antibodies. In control sections (A), P31-43-CY3 was visible only after 3-h pulse, but not after a 24 h chase. In the section of a celiac disease patient, P31-43-CY3 was present at both 3 h and 24 h in the epithelial cells of crypts. Overlay panels show that in cultivated biopsies from celiac patients at any time and in controls only at 3 h pulse, the P31-43 peptide co- localised with EEA1. White arrows indicates localisation of peptide in the early endocytic vesicles. Representative results from 3 independent experiments are shown.

We investigated whether P31-43 enters the cell and traced its localisation in intestinal biopsies from CD patients. For this set of experiments, we labelled P31-43 with CY3, a fluorochrome that is excited at 553 nanometers and emits at 575 nanometers. Intestinal biopsies were obtained from patients with CD and control subjects, kept in culture, and pulsed for 3 h with P31-43-CY3 (Figure 8A and B). In all biopsies, after 3 h pulse, peptide P31-43-CY3 was seen in epithelial cells, both in the crypts and in the villi (not shown) where it interacted with vesicles at the apical portion of the cells. Staining with anti EEA1 antibodies shows that the vesicles interacting with P31-43-CY3 are early endocytic vesicles. The overlay panels in Figure 8A and B show co- localisation of P31-43-CY3 and EEA1 in controls subjects and in patients with CD in the active phase of the disease. After 24 h chase, P31-43-CY3 was seen only in cells from CD patients. In this case the labelled peptide also interacted with the vesicular compartment and co-localised with EEA1 (Figure 8B). In normal controls, P31-43-CY3 entered the cells and interacted with EEA1-positive vesicles after a 3 h pulse, but the peptide was no longer seen after a 24-hours chase (Figure 8A). This indicates that, in healthy controls, this peptide is readily processed by the vesicular compartment. In the celiac environment, in which the peptide interacts with the vesicular compartment, P31-43 is delayed in early endocytic vesicles also at 24-hours chase.

## Discussion

In this paper we demonstrate that A gliadin peptides P31-43 and P57-68 enter CaCo2 cells. P31-43 localises on the endocytic membranes and delays vesicle trafficking by interfering with Hrs-mediated maturation of early endosomes in cells and enterocytes. Consequently, EGFR and possibly other receptors activation is extended with multiple effects on various metabolic pathways and cellular functions.

Although little is known about the processing of gliadin peptides, there is evidence that they enter enterocytes. [31–32–33]. Recently two papers [29]–[34] have described entrance and localization of P31-43 and P57-68 gliadin peptides, one localising P31-43 to the level of early endocytic vesicles using electro-microscopy, (which is consistent with our findings), and the other localising it to the level of the late vesicles using light microscopy of biotinylated peptides. Zimmer et al have shown, that P31-43, which is found in early vesicles, is not presented to stimulate gluten sensitive T-cells, in contrast P57-68 is found in late vesicles and can be presented in this manner. [29] The results of our experiments show that both P31-43 and P57-68 enter CaCo-2 cells and interact with the vesicular compartment. Their entrance is an active process that requires a temperature of 37C and Ca++ in the media. Methyl-Beta-Cyclodextrin, an inhibitor of endocytosis, prevents the entrance of both peptides indicating that they enter the cells by endocytosis. [35]–[36]

We mapped the distribution of P31-43 and P57-68 along the endocytic pathway using markers of early endosomes (EEA1; RAB5-EGFP) and late endosomes (LAMP 2; RAB7-EGFP). P57-68 could progress from the early, EEA1 positive, endocytic compartment to the late, LAMP2 positive, compartment after a 3 h chase. P31-43 instead interacted both at 30 minutes and 3 hours with the early endocytic compartment. Vesicular dynamic correlates with proper maturation of early endocytic vesicles [30] and can be altered by proline/glutamine rich proteins such as Huntingtin. [37] We therefore, investigated the motility of vesicles carrying P31-43-liss and P57-68-liss. Live observation of cells treated with fluorescent peptides (time lapse) indicated that the P31-43-carrying vesicles are slower than those carrying P57-68 at both 30 minutes and 3 hours. Taken together, these results suggest that P31-43 remains in the early endocytic vesicles, thereby delaying maturation of these vesicles into late endosomes by affecting endocytic motility. Moreover P31-43, but not P57-68 was able to delay endocytic vesicles containing EGF-Alexa [22] and dextran indicating that P31-43 interferes with vesicular dynamics no matter what cargo they are carrying. Consequently EGFR and other receptors can stay longer activated. There is in fact compelling evidence that endocytic membrane trafficking regulates signalling by extra cellular ligands. [38]

**Figure 9:**
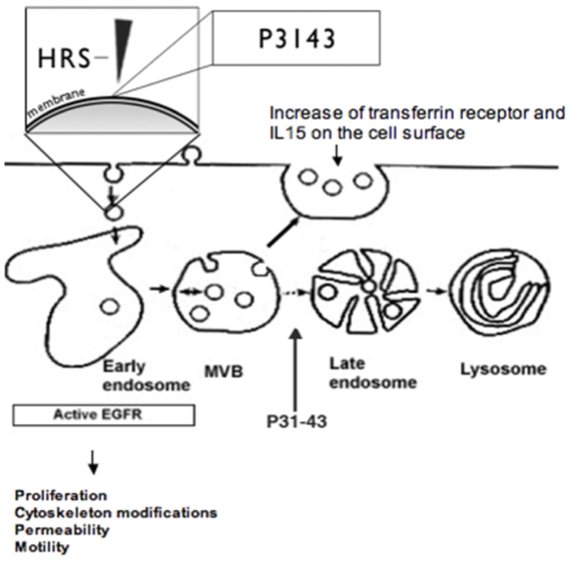
Overview of the effects of P31-43 on the endocytic pathway. Endocytosis has many effects on signalling: in fact, signalling pathways and endocytic pathways are regulated in a reciprocal manner. It is now widely accepted that the “Endocytic Matrix” is a master organiser of signalling, governing the resolution of signals in space and time. Consequently endocytosis affects several cell functions that range from proliferation to cell motility [50]. Growing evidences [22, 33, 34 and the present paper], point to an effect of certain gliadin peptides (i.e. P31-43) on the endocytic compartment. By interfering with Hrs localisation to the endocytic membranes, P31-43 induces two important effects: a) it delays endocytic maturation, and b) it alters the recycling pathway. By delaying the maturation of endocytic vesicles P31-43 reduces EGFR and other RTK degradation and prolongs their activation which in turn results in increased proliferation, actin modification and other biological effects. The alteration of the recycling pathway is able to direct more transferrin receptor and likely other recycling receptors such as IL15 to the membranes.

The delay of decay of the EGF receptor may have different consequences in different cell types because it affects several pathways and different functions (cell reproduction and survival, permeability, motility, endocytosis etc.) (Figure 9). [39]–[40]. We previously showed that gliadin peptides, and in particular P31-43, induce actin rearrangements and cell proliferation in various cell types, thereby mimicking the effect of EGF. Peptide 31–43 induces phosphorylation of EGFR and of the downstream effector signalling molecule ERK [22] which indicates activation of the EGFR pathway. Enhancement of the EGF pathway by gliadin and P31-43 is due to delayed inactivation of EGFR. [22].

It is likely that endocytic delay could also affect the innate immune response and cytokine metabolism. We have shown (MV Barone, unpublished data) that in CaCo2 cells gliadin peptide P31-43 can enhance the recycling endocytic compartment. As a consequence of this process, more transferrin receptor and IL15 accumulates on the cell surface. Recently, the recycling transferrin receptor has been implicated in the pathogenesis of CD. In fact, transferrin receptors are increased in celiac intestine and also function as IgA receptors that retrotranscytose P31-49 linked to IgA. [41] Taken together these data suggest that an important pathogenetic event in CD is the interference of gliadin peptide P31-43 with the endocytic compartment.

A data bank search using P31-43 as the query sequence, revealed strong sequence similarity with a region of Hrs, which is an important regulator of endocytic trafficking. Hrs is the main coordinator of endocytosis and signalling. It is part of a large complex, located to early endocytic vesicles and the multivesicular body, that is involved in the ubiquitination of proteins destined to lysosomes. It can be phosphorylated in cells treated with growth factors and cytokines [30] and is itself ubiquitinated. These post-translational modifications are needed for efficient sorting by Hrs of ubiquitinated membrane proteins to the degradation pathway. In cells where Hrs has been silenced, mutated or dislocated from the endosomes, EGFR and other receptor tyrosine kinases stay activated longer [42] and are recycled back to the cell surface. [43]

The sequence similarity between gliadin peptide P31-43 and Hrs involves a small area of the proline/glutamine-rich domain of the latter. Although gliadin is a well known proline/glutamine-rich protein, the homology of P31-43 with this Hrs domain is specific because the rest of the gliadin proline/glutamine-rich sequence does not share the same degree of similarity with Hrs. Moreover, P57-68, another gliadin peptide with a similar amino acid composition, does not produce the same effects in cells. This Hrs domain, at its COOH end, contains the clathrin-binding domain that brings clathrin to clathrin coated vesicles, [27] and is one of the domains needed to localise Hrs to the vesicle membranes. [44–45–46]

We have demonstrated in Hrs-EGFP transfected CaCo2 cells that P31-43, but not P57-68 co-localises with Hrs on the membrane of endocytic vesicles after 15 minutes of treatment, suggesting that the two peptides may have a different route to enter endocytic vesicles. Up to now no receptor has been found for P31-43 uptake. (Barone et al. unpublished results). Vilasi et al. [47] have proposed an alternative possibility investigating the interaction of the gliadin peptides with a very simple model of lipids micellae. They showed that P31-43 but not P57-68 can directly interact with the micellae, a good indication that it is possible for P31-43 to travel through the membranes and possibly reach the HRS molecules on the surface of the vesicles. We next evaluated whether P31-43 could interfere with Hrs localisation to the endocytic vesicles.

Western blot analysis of proteins extracted from the cell cytosol and membranes, together with immunofluorescence, showed that P31-43 treatment for 3 h, moved HRS from the vesicles to the cytosol. Furthermore, if P31-43 interferes with Hrs localization, it follows that a large excess of Hrs should prevent the proliferative activity of the gliadin peptide on cells. In fact, over expression of Hrs-EGFP prevented the effect of P31-43 on CaCo-2 proliferation. Taken together these results suggest that P31-43 interferes with Hrs-mediated maturation of early endosomes.

We also examined P31-43 trafficking in cultured intestinal biopsies from CD patients and controls using pulse and chase experiments. We show that P31-43 enters the enterocytes of cultured intestinal biopsies and localise, after a 3 h pulse, in early endocytic vesicles of enterocytes of intestinal biopsies from normal control subjects and non-treated celiac patients. However, after a 24-h chase, the peptide was still in the early endosomes of celiac enterocytes, but not in those of controls. This suggests that celiac patients are particularly susceptible to the effect of P31-43. We previously reported that endocytosis of EGF is delayed in enterocytes of atrophic celiac mucosa cultured *in vitro* with P31-43. [22] In the same context, P31-43 increased proliferation of crypts enterocytes – an effect that was prevented by EGFR inhibitors. [22]. Similar to the effects we observed in CaCo-2 cells, P31-43 probably delayed maturation of early endocytic vesicles also in cultured biopsies. This process prolongs EGFR activation and culminates in increased EGFR-dependent proliferation of crypt enterocytes as we have previously shown. [22] These observations suggest that the EGF pathway plays a central role in initiating and maintaining the high proliferation rates observed in the crypts of celiac patients. [48]–[49] This finding explains at least in part the role played by gliadin in remodelling of the celiac mucosa.

From a general point of view it is interesting to note that peptides from a very common alimentary protein, the gliadin, can have several metabolic effects due to the interference with important cellular functions, such as those regulated by the endocytic pathway. It remains to be established why P31-43 has a peculiar effect on the celiac intestinal mucosa. Celiac patients may have an alteration of the endocytic pathway (or some other related metabolic pathway) that renders cells more sensitive to the effect of P31-43 on endocytic maturation.

## Supporting Information

Figure S1 Control panel for EEA1 staining did not show any cross-excitation of fluorocromes: CaCo-2 cells, after 30 minutes pulse with P31-43-liss (red), were fixed permibilised and stained with an isotype matched primary antibody and anti-goat secondary antibodies Alexa-488 conjugated (green). The control shows that there isn't any cross-excitation of fluorocromes between the Alexa-488 conjugated secondary antibody and the lissamine linked to the peptide. Merge of the red and green panels is shown. The results are representative of 4 independent experiments.
(2.37 MB TIF)

Movie S1 In Materials and Methods of the text.
(1.88 MB AVI)

Movie S2 In Materials and Methods of the text.
(2.42 MB AVI)
