# Supplementary figures and images for: Gliadin Peptide P31-43 Localises to Endocytic Vesicles and Interferes with Their Maturation
Source: PLoS One. 2010 Aug 18;5(8):e12246. doi: 10.1371/journal.pone.0012246 (PMC2923621; doi:10.1371/journal.pone.0012246)

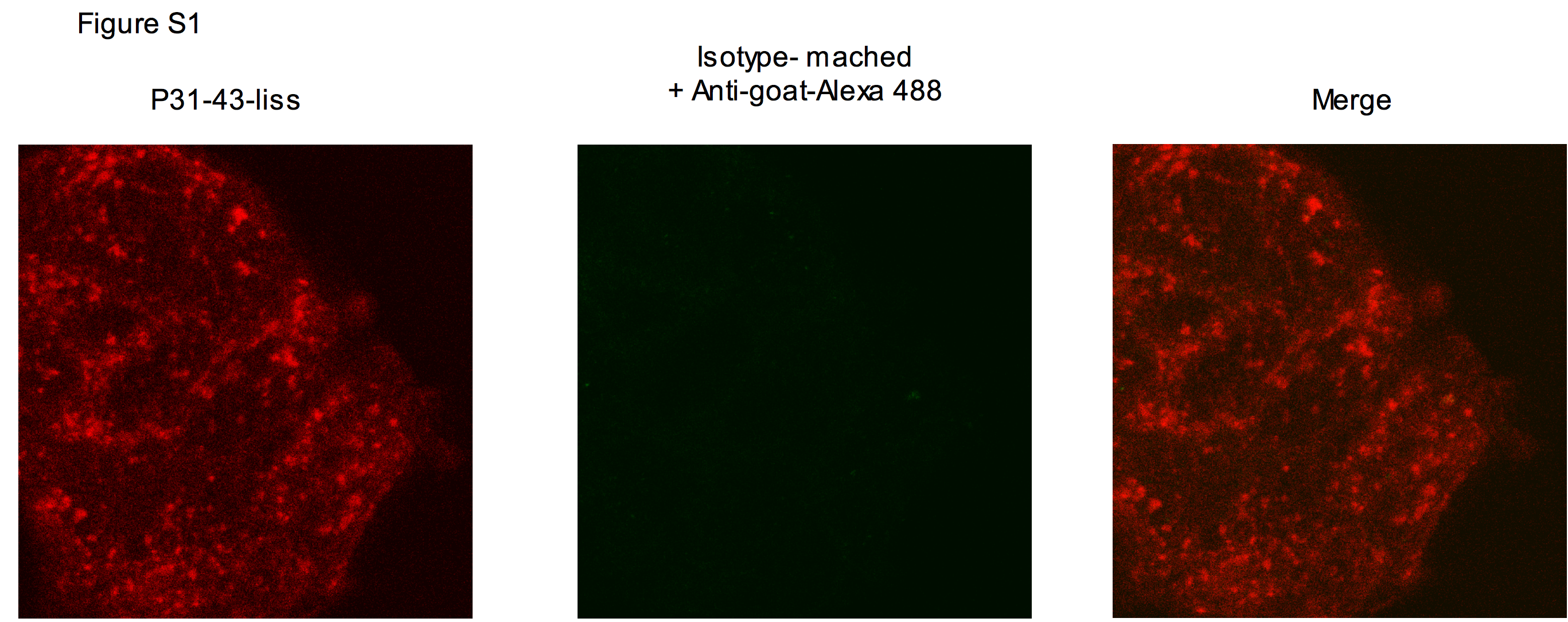

Supplement: Figure S1 — Control panel for EEA1 staining did not show any cross-excitation of fluorocromes: CaCo-2 cells, after 30 minutes pulse with P31-43-liss (red), were fixed permibilised and stained with an isotype matched primary antibody and anti-goat secondary antibodies Alexa-488 conjugated (green). The control shows that there isn't any cross-excitation of fluorocromes between the Alexa-488 conjugated secondary antibody and the lissamine linked to the peptide. Merge of the red and green panels is shown. The results are representative of 4 independent experiments. (2.37 MB TIF) [file pone.0012246.s001.tif]
